# Real-Time Gender Recognition for Juvenile and Adult Faces

**DOI:** 10.1155/2022/1503188

**Published:** 2022-03-17

**Authors:** Sandeep Kumar Gupta, Seid Hassen Yesuf, Neeta Nain

**Affiliations:** ^1^Department of Computer Science and Engineering, Malaviya National Institute of Technology, Jaipur-302017, Rajasthan, India; ^2^Department of Computer Science and Engineering, University of Gondar, Maraki 196 Gondar, Ethiopia

## Abstract

Facial gender recognition is a crucial research topic due to its comprehensive use cases, including a demographic gender survey, visitor profile identification, targeted advertisement, access control, security, and surveillance from CCTV. For these real-time applications, the face of a person can be oriented to any angle from the camera axis, and the person can be of any age group, including juveniles. A child's face consists of immature craniofacial feature points in texture and edge compared to an adult face, making it very hard to recognize gender using the child's face. Real-word faces captured in an unconstrained environment make the gender prediction system more complex to identify correctly due to orientation. These factors reduce the accuracy of the existing state-of-the-art models developed so far for real-time facial gender prediction. This paper presents the novelty of facial gender recognition for juveniles, adults, and unconstrained-oriented faces. The progressive calibration network (PCN) detects rotation-invariant faces in the proposed model. Then, a Gabor filter is applied to extract unique edge and texture features from the detected face. The Gabor filter is invariant to illumination and produces texture and edge features with redundant feature coefficients in large dimensions. Gabor has drawbacks such as redundancy and a large dimension resolved by the proposed meanDWT feature optimization method, which optimizes the system's accuracy, the size of the model, and computational timing. The proposed feature engineering model is classified with different classifiers such as Naïve Bayes, Logistic Regression, SVM with linear, and RBF kernel. Its results are compared with the state-of-the-art techniques; detailed experimental analysis is presented and concluded to support the argument. We also present a review of approaches based on conventional and deep learning methods with their pros and cons for facial gender recognition on different datasets available for facial gender recognition.

## 1. Introduction

Gender prediction from the face data has considerable attention due to wide application and use cases compared to other attributes such as age, emotion, and ethnicity. Facial gender classification has real-time commercial applications to determine a criminal's sex in non-invasive forensic, a restricted entry for specific sex and specified zones such as website or woman clubs, surveillance of particular gender, law enforcement, human-computer interaction, and access control. For commercial applications, gender prediction is used for sex demographics in the crowd from CCTV, real-time gender-specific targeted marketing, advertising of a product for a specific sex group, and any access segregation based on gender. For example, train compartments (or seats), metros, buses, washrooms, and hostels have restricted access to a specific gender in some countries where the passengers or visitors based on sex can be monitored for any law violation of this access. Gender recognition helps collecting customer and visitor demographic statistics in the business zone or public zone (banks, malls, airports, and railway stations) for better planning and effective business strategies. Specific gender-targeted advertisements and recommended systems change the promotions automatically on an electronic board targeting a particular gender. A machine interview system is demanded to recognize a person's face as gender during physiological behavior analysis in human-computer interaction. In CCTV surveillance and security, auto gender recognition is needed to monitor permitted zones for a particular gender. In biometric systems, gender recognition can be used to reduce the search index of a database. During the COVID-19 pandemic, the accuracy of face recognition application is reduced due to wearing a mask on the face, but gender recognition can be used to increase the accuracy as a covariate in this case. The facial gender prediction methods can be grouped into conventional learning and deep learning-based approaches. In the conventional learning approach, handcrafted features are extracted to represent gender patterns from face images. In the deep learning approach, a convolutional neural network (CNN) and its different versions extract texture features to determine gender from a large facial image set by statistical training with strong nonlinear modeling ability. The drawback of the deep learning-based approach (CNN) is that it requires a vast amount of training data (here, face images) and huge computation time for model regularization. Furthermore, when the network is affected by noise, overfitting is possible and underfitted if fewer face images are used. In a constraint environment, both approaches effectively represent gender prediction. The gender pattern is highly correlated with the face's texture features such as wrinkle depth, texture relationship, beard, eyebrows, and lip shape in the adult faces, while juvenile faces consist of skin texture, eyebrows, and lip shape as primary feature space for gender prediction. Furthermore, in juveniles (age group 2–8 yrs), it is challenging to identify the gender accurately by human intelligence too. Teru and Chakraborty [1] experimented and concluded that the accuracy of gender recognition from the face image is affected by factors such as illumination, orientation, and noise in an image. The pose of the face specifies the face angle related to the camera. The face can move in three different directions: (a) roll (in-plane rotation), (b) yaw (left-right rotation), and (c) pitch (up-down rotation) [2]. If the face detection accuracy decreases due to illumination and rotation variation, it also affects the facial gender identification accuracy. Facial emotion, ethnicity, age, and occlusions of the face, such as facial hair, eyeglasses, and hats, also impact gender recognition accuracy. The above facts are also mentioned in the literature; Abdelkader and Griffin [3] experimented on a large set of 12,964 facial images and found that an improper broad number of elderly females and young males were falsely classified. After an empirical study of several facial gender recognition methods, Guo et al. [4] concluded that the accuracy of gender prediction is influenced significantly by a person's age; faces of adults have higher accuracy than young.

In general, applications of facial gender are limited primarily up to the adult population. It is needed to include juvenile faces also where facial gender recognition is hard to recognize correctly due to immature craniofacial features on children's face [5, 6]. The research on juveniles also faces not scaling too much due to the limited availability of the dataset. The uncontrolled dataset's juvenile face includes real-life challenges such as illumination, rotation of face head, scale variation, and obstacles. It makes the performance of gender recognition adversely. Facial gender in juvenile face data with the above challenges has vital applications such as the finding of missing child/suspect juvenile [7] with other facial attribute recognition systems; finding of prohibited uploading images and videos of sexually abused specific juvenile gender [7]. To address the above challenges, the UTK dataset [8] is used, which has juvenile faces with some uncontrolled environmental challenges. The contributions of this paper are as follows:

- For compact and minimal redundant feature vectors, a new feature engineering is proposed and evaluated on various classifiers such as (a) support vector machine with the linear kernel (SVM-linear), (b) SVM-radial basis function (RBF) kernel, (c) Logistic Regression, and (d) Naive Bayes.
- Real-time system: in this paper, a comprehensive design for gender prediction in real time is proposed. The real-time problem of gender identification includes the orientation of the person's head captured in the camera, which is solved using a progressive calibration network (PCN) face detector.
- High accuracy: the proposed system provides a high level of accuracy.
- Robust: the system's accuracy is consistent with real-life challenges such as variation in pose, illumination, profile, background change, expression variation, obstructions due to wearing a hat, glasses, face with a beard hairstyle, and all age groups, including juveniles faces where gender recognition is hard.

The rest of the paper includes the following sections: Section 2 describes the related development and comparative work carried out in this facial gender recognition from the initial stage; Section 3 includes the proposed architecture; Section 4 includes different results of experiments on the dataset; analytical discussion is followed by Section 5 for deriving the conclusions and identification of possible future gaps.

## 2. Related Work

The related work of facial gender classification is segregated into two parts: (a) dataset available for studies of the facial gender classification and (b) different approaches of gender classification carried out by researchers till date. The related work is described in the following two subsections.

### 2.1. Facial Dataset for Gender Recognition

The facial dataset is needed to experiment and benchmark result performance on different parameters for gender recognition. A real-time face dataset for gender recognition must include a large sample size of images with different subjects, balanced gender ratio, race distribution, age variations, and real-life environment variation to examine the facial gender recognition (FGR) system. There are huge real-life factors such as Resolution (R), Scale (s), Hair (H), Race (R), Longitudinal (L), Illumination (I), Uncontrolled Environment (U), Controlled Environment (C), Frontal View (F), Profile (P), Occlusion (O), Expression (E), and Sharpness (S). For gender prediction, the most commonly used datasets and their properties are shown in Table 1. The dataset category can be divided into two parts: (a) controlled dataset and (b) uncontrolled dataset. The controlled dataset is collected in a specific condition only, but the uncontrolled dataset includes all different real-life challenges.

**Figure 1:**
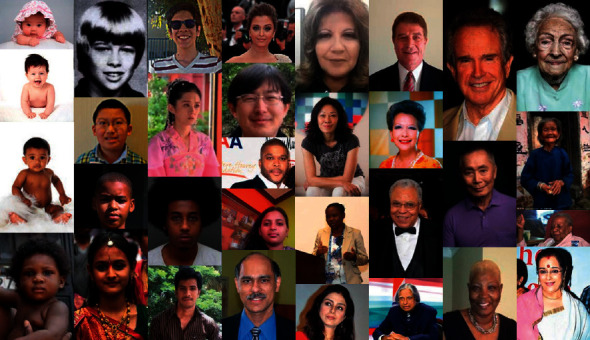
Sample of UTK Face dataset which includes the age range 0–116 with different ethnicity, facial expression, illumination, occlusion, and orientation.

FERET dataset [12] is a widely used dataset for facial gender recognition in a controlled environment having a better resolution compared to MORPH and FG-NET. It has adult gender information, profile variation, and more representative texture information, making it better for extracting local descriptors. FERET contains 14,051 images of 512 × 768 pixel resolution in the PPM format for adult faces. UTK cropped Faces have 20,000 images for different age groups from 0 to 116 years, with cropped images of resolution size of 200 × 200 pixels. Samples of the UTK dataset are shown in Figure 1. Both UTK Face and FERET datasets [12] are challenging due to variations in rotation, POSE (position, orientation, scale, and expressions), background, illumination, age groups, hairstyle, and obstructions as spectacles, bandage, scarves, cap, birthmarks, eye color, moles, and cuts. The UTK Face dataset is more challenging due to juvenile and immature faces with texture patterns of sex variability than adult faces. This real-life variability of the UTK Face dataset [8] and the FERET dataset [12] makes it hard to classify facial gender correctly in these types of images. Labeled Faces in the Wild (LFW) [10] dataset contains facial images captured in an unconstrained environment. It has significant daily-life variations, such as lighting, pose, background, accessories, race, and occlusions. It contains images of celebrities and politicians with repeated individuals. IMDB-WIKI [13] dataset has real-world challenges such as sketch faces, poor quality, and human comic faces. It includes blank images also, which influence the network prediction in a contrary manner. CelebFaces Attributes (CelebA) dataset has a wide range of facial attributes containing more than 200K images of celebrities. The Adience dataset [14] consists of gender and relative age labels divided into 5 folds. It collects Flickr images with variations of illumination, pose, image quality, and noise, making it harder to recognize correctly. MORPH dataset [9] is divided into different albums. It consists of the acquisition date, birth date, ethnicity, and gender information. FG-NET [11] dataset consists of 1,000 images of 82 individuals prepared through scanning photos. It has variations in background, illumination, and resolution.

### 2.2. Analysis of Related Work for Facial Gender Recognition

**Figure 2:**
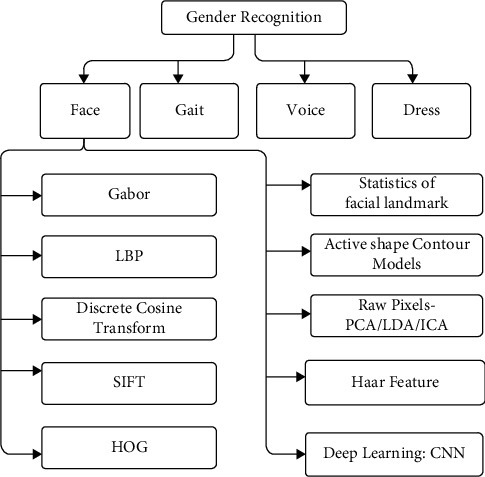
Brief classification of the state-of-the-art work for gender classification.

Facial gender recognition (FGR) is categorized into (a) conventional learning and (b) deep learning-based approach. Gender recognition through conventional learning includes handcrafted feature engineering. The model is trained on handcrafted features, and classification is performed on a test dataset. A convolutional neural network is used for feature extraction and classification in the deep-learning approach. Deep learning has constraints such as requiring a large quantity of data for model fitting on high computation machines such as graphical processor units (GPUs) during training. For juvenile gender recognition, fewer images are available in the public dataset. Compared to deep learning, the conventional learning approach can be regularized even for a limited dataset. This study analyzes the methods of gender recognition developed so far from 2001 to 2021. The review is consolidated in Table 2 and followed by a detailed analysis. Different traits such as gait, face, voice, fingerprint, and dress of a person can be used to identify gender, accepted from the literature shown in Figure 2. The face is the most suitable for gender prediction from these data due to its easy visibility, availability, collectability, acceptability, and universality. Table 2 shows various methods of gender prediction such as the Gaussian mixture model (GMM), scale-invariant feature transform (SIFT), discrete wavelet transform (DWT), discrete cosine transform (DCT), histogram of the gradient (HOG), local binary pattern (LBP), dimension reduction techniques, such as Haar features, active contours, statistics features, independent component analysis (ICA), principal component analysis (PCA), and deep learning techniques. These methods and their different improved versions with various classification techniques have been proposed so far. The various techniques of conventional feature engineering include texture-based methods such as local binary pattern (LBP), histogram of gradient (HOG); Haar-based features; feature separation techniques such as discrete cosine transform (DCT). Based on feature space, the feature extraction of gender identification from face images is classified into two approaches: geometric (local features) and appearance (global features)-based feature extraction. In the geometric approach, parts of the face, such as the mouth, eyes, lips, and nose, are considered as the feature space, while in the appearance-based approach, the whole face is the feature space. Leng and Wang [16] used the Gabor filter to extract edge details. The SVM classifier for gender recognition achieved 98% accuracy on frontal adult faces in a controlled environment. Wang et al. [20] proposed scale-invariant facial gender recognition with an AdaBoost classifier and obtained 97.0% accuracy. Rai and Khanna [17] extracted features using the Gabor filter where the dimension of Gabor features is reduced using 2D PCA with the SVM classifier achieving a better accuracy of 98.18% on the FERET dataset. Mohamed et al. [19] used DWT and DCT feature extraction techniques with the SVM classifier which outperforms with 95% accuracy on the FERET dataset compared to [18] which used DWT feature extraction with SVM classification and obtained 92% accuracy and [21] used DWT and PCA for feature extraction, fisher discriminant analysis (FDA) for classification and achieved 95% accuracy. ICA feature extraction [24] with linear discriminant analysis (LDA) classification obtained the higher accuracy of 99.3% on the FERET dataset compared [23] with 85% accuracy by using PCA feature extraction and LDA classification for gender prediction. On the one hand, Tapia and Perez [25] used various spatial scale feature fusions, which is selected using intensity, mutual information from shape, and histogram of LBP, where gender classification is performed by SVM and obtained 99.1% accuracy. On the other hand, Makinen and Raisamo [26] used LBP, Haar features, and SVM classifier and achieved 92.86% accuracy. In contrast, [27] they applied the nearest neighbour classifier on the LBP feature to achieve 98.82% accuracy. Moeini et al. [28] introduced a model with local gabor binary pattern (LGBP) features and SVM classifier and obtained 98.55% accuracy. In [29], 93.92% accuracy is achieved with compass local binary pattern (LBP) features and SVM classifier while Annalakshmi et al. [30] obtained 97.61% accuracy with the histogram of gradient (HOG) and spatially enhanced local binary pattern (SLBP) features with SVM classifier. Afifi and Abdelhamed [32] used a combination of the facial components called foggy face to extract features and CNN for classification and achieved 99.28% accuracy. By comparing conventional learning approaches in Table 2, the best accuracy is achieved by [25] because they used feature fusion at various scales, which improves the performance of gender classification. Comparing the conventional learning approach with the deep learning approach, better accuracy is gained by deep CNN. Gender analysis is carried out on the UTK Face dataset using conventional and eep Learning approaches. Embedding face similarity depends on both facial components and face similarity. Two men's faces are more similar than men's and women's faces because face embedding (Euclidean distance) between two men's faces is more minor than the Euclidean distance of face embedding a man and a woman. Same-gender faces are grouped due to facial embedding, so k-nearest neighbor (KNN) also gives higher accuracy 97.02% on the UTK Face dataset for gender recognition compared to logistic regression with 92.4%, SVM with 88.4%, Naive-Bayes classifier with 89.4%, and decision tree with 93.2% accuracy [33]. Teru and Chakraborty [1] used 3 classifiers for gender recognition. Fader CNN uses a 3 × 3 size kernel, one padding, and a stride of 2, where ReLu and batch normalization follow every CNN layer. Dropout is applied to the first, third, and fourth layer of convolution and the fully connected (FC) layer. The accuracy achieved on the UTK Face dataset was 84.83%. Simple CNN that contained the same topology and trained on latent representation obtained 89.51% accuracy. Simultaneously, CNN-WL is the CNN with weighted loss which obtained 88.80% accuracy on the same dataset. Song and Shmatikov [34] used CNN to classify gender with overlearning sensitive attributes and achieved 90.38% accuracy on the UTK Face dataset. Bragman et al. [35] applied a stochastic filter group in CNN architecture for gender recognition and obtained a 92.46% recognition rate. Nagpal et al. [36] used CNN in which filter drop is applied before fully connected layer to classify gender, and 94.60% accuracy is achieved on the UTK Face dataset. Das et al. [37] introduced multitasking CNN, which used dynamic joint loss for gender recognition on the UTK Face dataset and obtained an 88.80% recognition rate better than the conventional and deep learning technique used for gender recognition on the UTK Face dataset.

State of the art with other datasets is as follows: Hassner et al. [39] used LBP, FPLBP, and SVM to achieve 79.3% accuracy on the Adience dataset in which images are captured in the uncontrolled environment, including all age groups. This dataset contains children with fewer distinct facial features and is difficult to recognize in an unconstrained environment. Results demonstrate that LBP is suitable for constraint images. Khan et al. [40] used conditional random fields (CRFs) to segment the face into 6 classes (hair, skin, mouth, nose, eyes, and back). CRF uses different facial parts hierarchy and mutual relationship between them. Shape, color, and position features are extracted using CRFs. Then, the probability map is generated for each class used as a gender descriptor and given to a random decision forest classifier (RDF). It used the Adience dataset and achieved 91.4% accuracy for the same.

## 3. Proposed Work

**Figure 3:**
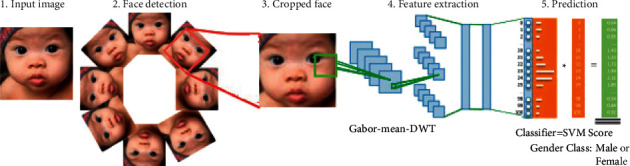
Block architecture of the proposed model for facial gender identification for uncontrolled-oriented juvenile faces. The phases of proposed model consists of (a) orientation invariant face detection (PCN network), (b) feature engineering on detected face using Gabor features, (c) feature reduction using meanDWT, and (d) classification.

The proposed work includes a complete pipeline including problem formulation and the proposed solution from image acquisition to classification for gender identification. The training and testing face images are downloaded from a respective repository of the UTK Face dataset and the FERET dataset. The architecture with a brief pipeline of phases of the proposed model is presented in Figure 3. The face detection is applied to the above images using PCN face detection to detect rotated faces. The proposed feature extraction approach using the Gabor-mean-DWT model is designed and applied. The output of feature extraction with labeled classes is applied for learning (converge the network during training) the model with any one of classifiers: (a) support vector machine (SVM-Linear), (b) support vector machine-radial basis function (SVM-RBF), and (c) logistic regression (LR).

### 3.1. Problem Statement

A facial image set *I*=*I*_*i*_ ∈ *R*^*m*×*n*^ is given, where *i*=1,2,  . ., *N*, and each image *I*_*i*_ having the ground-truth label of gender (*y*_*i*_) ∈ *Y*. Here, *Y* ∈ *R*^*N*×1^ is the set of ground truths respective to image *I*, and *N* is a number of images set *I*. The objective function *F* of problem is the evaluating probability for the gender class (male or female) defined as $\hat{y}=F\left(I_{i}\right)$ for the given test image set *I* such that the mean average of difference between the predicted class $\left(\hat{y}\right)$ and the actual class (*y*) must be minimized for increasing the absolute accuracy (*A*) over the image set (I) defined as *A*(*I*) in the following equation:

(1) $$\begin{array}{c} A\left(I\right)=1-\frac{1}{N}\sum_{i=1}^{N}\left|\hat{y_{i}}-y_{i}\right|. \end{array}$$

For real-time gender recognition, a facial image is affected by different real-time factors such as profile, orientation, scale, and illumination. These factors reduce face detection accuracy, which subsequently reduces the overall application of gender recognition. The problem of the oriented face is detected correctly using orientation invariant face detection. Here, the PCN network is used for orientation in invariant face detection. A real-time facial gender classification model includes (a) image acquisition from vision sensor (camera), (b) face detection in captured frame, (c) image prepossessing to eliminate the noise on the detected face and enhancing the image for feature engineering, (d) feature engineering for feature generation such as texture, edge, and subsequently, feature selection, and (e) gender prediction. The accuracy (P) of facial gender classification in the natural environment depends on all the above phases, primarily face detection, feature extraction, and classification. The proposed model's accuracy is improved compared to other state-of-art techniques using optimization on phases of face detection using the orientation invariant method on feature engineering using illumination invariant feature extraction and optimized feature selection, and the use of state-of-art classifiers for gender recognition is explained in the following subsections. The block diagram of the complete process is shown in Figure 3.

### 3.2. Orientation Invariant Face Detection

Face images acquired in real-time by CCTV, mobile, or any other vision sensors are nonfrontal. Frontal face detection affects the accuracy of detected faces and subsequently reduces the accuracy of facial gender recognition for real-time scenario. Suppose the orientation invariant face detection method is used in this stage for a real-time scenario. In that case, it enhances the feature extraction stage of FGR to identify the unique gender features. For this problem, the progressive calibration network (PCN) face detector [42] is applied, which detects faces with various orientations in fame. In PCN, the image pyramid principle is used to evaluate each sliding window's score (probability of a face). For a low confidence score, nonface sliding windows are rejected simultaneously. Face detection is performed using the concept of three-stage cascaded calibration. The objective function *f*_1_ of stage-1 PCN face detector for image *I*_*i*_ is shown in (2) with the overall loss function *L* evaluated as

(2) $$\begin{array}{c} \left[s,r,c\right]=f_{1}\left(I_{i}\right), \end{array}$$

where *s*, *r*, *c* represent the face bounding box's confidence score, coordinate vector of the bounding box, and score of orientation angle. Equation (3) shows the overall objective function as loss *L* of PCN:

(3) $$\begin{array}{c} L=\min\left(L_{s}+\lambda_{r}L_{r}+\lambda_{c}L_{c}\right). \end{array}$$

Here, *λ*_*r*_, and *λ*_*c*_ are the loss balance parameters for regression and calibration, respectively. *L*_*s*_, *L*_*r*_, *L*_*c*_ are the loss functions of three parameters (*s*, *r*, *c*) of PCN objective function *f*_1_. The overall minimization of loss *L* needs to minimize the three different parameters *L*_*s*_, *L*_*r*_, *L*_*c*_ which are evaluated in the following equations:

(i) Classification of face and nonface in moving sliding windows (*W*_*i*_): it is the classification process based on softmax score (*s*) where the loss function *L*_*s*_ is presented in as
  (4) $$\begin{array}{c} L_{s}=y\;\log\;s+\left(1-y\right)\log\left(1-s\right),\\ Here,y=\left\{\begin{array}{cc} 1, & \text{if }{\text{W}}_{\text{i}}\text{ have the face},\\ 0, & \text{otherwise}. \end{array}\right. \end{array}$$
(ii) Second parameter of objective function is regressing the fine bounding box *r* for evaluating best coordinates of the bounding box to locate the face in the image as represented in the following equation:
  (5) $$\begin{array}{c} L_{r}\left(p,p^{*}\right)=T\left(p-p^{*}\right). \end{array}$$
Here, *p* is predicted, and *p*^*∗*^ is the original regression result. *T* is the cross-entropy loss.
(iii) Last parameter of the objective function *f*_1_ is the evaluating calibration score *c* (orientation of face) as represented in the following equation:
  (6) $$\begin{array}{c} L_{c}=y\;\log\;c+\left(1-y\right)\log\left(1-c\right),\\ y=\left\{\begin{array}{cc} 1, & \text{if }{\text{W}}_{\text{i}}\text{ is facing up},\\ 0, & \text{if }{\text{W}}_{\text{i}}\text{ is facing down}. \end{array}\right. \end{array}$$

After optimizing (3), the top confidence face is achieved by rejecting the maximum nonface sliding windows. The leftover face candidates are updated with a newly regressed bounding box and rotated with a predicted angle *θ*_1_ as defined in equation (7). For example, if the face is at *θ*_1_=0°, then no rotation is required, while *θ*_1_=180° means the candidate's face is facing down, so 180° rotation will make it face up.

(7) $$\begin{array}{c} \theta_{1}=\left\{\begin{array}{cc} 0^{{}^{\circ}}, & c\geq 0.5,\\ 180^{{}^{\circ}}, & c<0.5. \end{array}\right. \end{array}$$

In the next stage, the angle range is reduced to half, i.e., from [−180°, 180°] to [−90°, 90°], to find the precise orientation as defined in equation (8). The non-confident sliding windows are discarded, and head pose orientation is calibrated. The orientation range for the head position is predicted by classifying the candidate facing as up and down among three classes of rotation-in-plane (RIP) angle, i.e., [−90°, −45°], [−45°, 45°], or [45°, 90°] as defined in the following equation:

(8) $$\begin{array}{c} \theta_{2}=\left\{\begin{array}{cc} -90^{{}^{\circ}}, & \text{if }{\text{c}}_{\text{0}}\text{ is maximum score},\\ 0^{{}^{\circ}}, & \text{else if }{\text{c}}_{1}\text{ is maximum score},\\ 90^{{}^{\circ}}, & \text{else }{\text{c}}_{2}\text{ is maximum score.} \end{array}\right. \end{array}$$

Here, *c*_0_, *c*_1_, and *c*_2_ are the predicted scores of respective classes of orientation at the second stage. The candidates of the face should be rotated by −90°, 0°, or 90° as a par identified class. In the final stage, the range of RIP angle is reduced again by half, i.e., from [−90°, 90°] to [−45°, 45°], to evaluate the final precise angle of head orientation. In this stage, the classification of angle (*θ*_3_) is again performed in a precise range, i.e., [−45°, 45°], and the final head orientation is calibrated (*θ*_3_ ∈ [−45°, 45°]). The face predicted with the final calibrated angle (*θ*=*θ*_1_+*θ*_2_+*θ*_3_) is cropped $\left(\hat{I}\right)$ from input image *I* and rotated at 360 − *θ* angle to make a face horizontal for the next processing phase of feature engineering.

### 3.3. Feature Engineering Model

Cropped face $\left(\hat{I}\right)$ with the horizontal head position after processing face detection is passed into the next phase feature engineering model to extract texture and edges as key features from the cropped face to determine gender. The Gabor filter is helpful for edge and texture feature extraction from an image $\left(\hat{I}\right)$. It is a linear bandpass filter having the property of optimal localization in both frequency and spatial domains. The relevant frequency spectrum is captured by the Gabor filter to extract features at specified orientations for finding the above discriminating features from the image. The general function of the bidimensional Gabor filter is given by

(9) $$\begin{array}{c} \Psi \left(\theta ,\lambda ,\gamma ,\phi \right)=\exp\left(-\frac{{a^{\prime }}^{2}+\gamma^{2}{b^{\prime }}^{2}}{2\sigma^{2}}\right)e^{j\frac{2\pi a^{\prime }}{\lambda }}, \end{array}$$

where the Gabor filter (Ψ) has the following characteristics: *θ* denotes the orientation, which specifies the number of cycles/pixel, *λ* denotes the wavelength) offset of the sinusoid, *γ* is the angle of the normal to the sinusoid plane, and *ϕ* denotes the phase, respectively. Gabor is a product of a Gaussian sinusoidal, which has a property of illumination invariant. Gabor filter kernel (Ψ) [43] is defined in (9) which is evaluated using the projection angle *θ* and the direction coefficient *a*′ and *b*′ as defined in the following equation:

(10) $$\begin{array}{c} \begin{array}{c} a^{\prime }=a\;\cos\;\theta +b\;\sin\;\theta \\ b^{\prime }=-a\;\sin\;\theta +b\;\cos\;\theta \end{array}. \end{array}$$

Gabor feature matrices $\left(\hat{I_{\psi }}\right)$ can be generated through the convolution (*∗*) of the face image $\hat{I}\left(x,y\right)$, and Gabor filter kernel (Ψ) is defined as

(11) $$\begin{array}{c} {\hat{I}}_{\Psi }\left(x,y\right)=\hat{I}\left(x,y\right)*\Psi \left(\theta ,\lambda ,\gamma ,\phi \right). \end{array}$$

*ψ*(*θ*, *λ*, *γ*, *ϕ*) is the complex number in nature, as defined in (12), so Gabor feature is the addition of square after product of real kernel (*ψ*_*R*_) and imaginary components (*ψ*_*Im*_) with facial Image $\left(\hat{I}\right)$, which is shown in the following equation:

(12) $$\begin{array}{c} \Psi \left(\theta ,\lambda ,\gamma ,\phi \right)=\Psi_{R}\left(\theta ,\lambda ,\gamma ,\phi \right)+\Psi_{Im}\left(\theta ,\lambda ,\gamma ,\phi \right), \end{array}$$

(13) $$\begin{array}{c} {\hat{I}}_{\Psi }\left(x,y\right)=\sqrt{\hat{I}*\Psi_{R}{\left(\theta ,\lambda ,\gamma ,\phi \right)}^{2}+\hat{I}*\Psi_{Im}{\left(\theta ,\lambda ,\gamma ,\phi \right)}^{2}}. \end{array}$$

**Figure 4:**
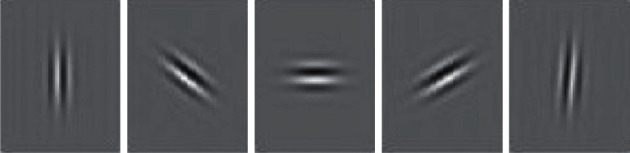
The theta controls the orientation of the Gabor function where zero degree corresponds to the vertical position of the Gabor function. Gabor Kernel represented at 5 angles *θ*={0,45,90,135,180}, respectively, (from left to right). *θ* changes the angle of Gabor kernel as zero degree represents a vertical edge and ninety degree of a horizontal edge.

**Figure 5:**
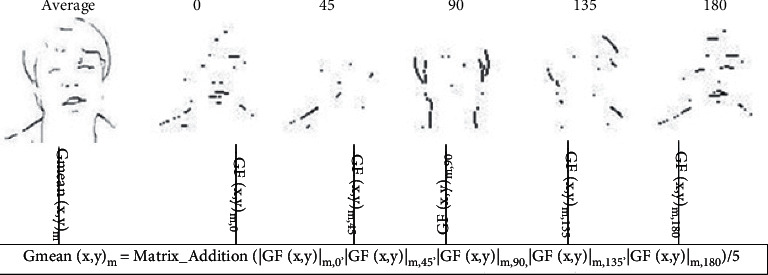
The 1^*st*^ face shows the cumulative mean features in a single matrix which reduce the feature dimensions and redundancy successfully without losing any information while remaining 5 faces show the facial features generated through Gabor kernel respective to 5 angles *θ*={0,45,90,135,180} on a sample face of the FERET dataset.

For generating Gabor edge-texture features using different orientation (*θ*), the Gabor (*ψ*) is applied from equation (9) to equation (13) on the detected face $\left(\hat{I}\right)$. Here, 5 Gabor orientations of *θ*=(0,45,90,135,180) are used for extracting the Gabor features from different orientations for each resolution scale *s*=(0.25, 0.5, 1,1.25, 1.5), as shown in Figure 4. The Gabor filter with 5 orientations and 5 scales generates 5 × 5=25 Gabor feature matrices. This high-dimensional feature space is one disadvantage of using a Gabor filter. The generated Gabor edges for scale *s*=1 and at different orientation 0,45,90,135,180 are shown in Figure 5 which represents the redundant edge pattern on the neck, eye, face corner, lips, and nose. This redundancy is another problem in Gabor filter-based feature extraction. We overcome this using proposed average-DWT feature engineering as defined in the next section.

#### 3.3.1. Gabor-AveragePool Feature Extraction

The Gabor feature matrix of different orientations for a fixed scale has the same dimension. These matrices are then added to generate a single matrix. Each coefficient of this matrix is divided by the number of angles. This way, the resultant coefficient is an average value of its position at different orientation coefficients ${\hat{I}}_{m}\left(i,j\right)$, as represented in equation (14). Therefore, it represents the 5 Gabor feature coefficient and reduces the dimensions by a factor of 1/5.

(14) $$\begin{array}{c} {\hat{I}}_{m}\left(i,j\right)=\frac{1}{5}\sum_{\theta =0:45}^{180}{\hat{I}}_{\psi }{\left(i,j\right)}_{\theta }. \end{array}$$

Here, *θ*={0,45,90,135,180}. Figure 5 shows the different edge and the texture coefficients for a sample face image according to different Gabor kernel for *θ*={0,45,90,135,180} and the results of Gabor-mean step. Some edge features show redundancy (shoulder edge, mouth edge, etc.) in different Gabor angle feature matrices in this figure. In contrast, resultant Gabor-mean shows a single average feature value. The proposed model using Gabor-mean reduces the dimension and redundancy, as shown in Figure 5. The first image of Figure 5 is represented by the average coefficients of the remaining five images respective to 5 orientations. It represents the unique pattern of facial gender in compact and concise features. The number of Gabor-mean coefficients is irrespective of the Gabor kernel of the angle used, regardless of existing Gabor filter-based approaches such as Gabor-DWT, Gabor-DCT, and Gabor-PCA.

#### 3.3.2. DWT Feature Extraction Technique on Gabor-AveragePool Features

Two-dimensional (2D) discrete wavelet transform (DWT) is applied on the extracted feature matrix ${\hat{I}}_{m}$ from the Gabor-AveragePool process. It is like the translation and dilation of a scaling function on facial images consisting of the low-pass filter (L) and high-pass filter (H).

**Figure 6:**
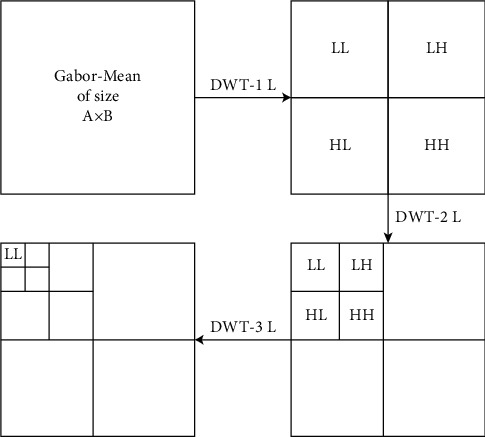
Block diagram of process of DWT feature engineering from Gabor-mean feature matrix.

DWT (2D-DWT) can be evaluated using the first one-dimensional (1D)-DWT on rows of a 2D image matrix. The same is evaluated on the columns of evaluated 1D-DWT. Here, the approximation, horizontal, vertical, and diagonal frequency blocks are represented by LL (low frequency), LH, HL, and HH (high frequency), as shown in Figure 6. The LL block represents the approximation of an image in a low dimension, while another block represents the details of the image. The low-frequency block (LL) has the property of smoothing of the input image [44]. A *k* level (scale) of the 2D-DWT process converts the input matrix (block) of size *M* × *N* sizes into 4 sub-blocks (LL, LH, HL, and HH), each having a size of *M*/2^*k*^ × *N*/2^*k*^. Here, we have used three scale/level of 2D-DWT iteratively on the LL block, and the final third level LL sub-block is extracted as the feature vector *X*  =  [*x*_1_, *x*_2_,…, *x*_*n*_], where *n* is the size of the feature vector. These features *X* are passed to the classification process for the input image *I*_*i*_. The block architecture of three level-DWT feature extraction on Gabor-mean is shown in Figure 6.

### 3.4. Classification Model

Extracted features *X* using proposed Gabor-meanDWT are passed to the classifier for training the network for convergence using the training set, and test data results are evaluated on the converged model. We have experimented on different classifiers such as (a) support vector machine (SVM) with the linear kernel (SVM-linear) and (b) SVM-radial basis function (RBF) kernel.

### 3.5. Support Vector Machine

Support vector machine (SVM) is the principle of structural risk minimization (SRM) where the objective is to find the best hyperplane that separates classes in input space and maximizes the margin between classes [45]. For the given training set (*X*_*i*_, *y*_*i*_), where *X*_*i*_=*x*_1_, *x*_2_,…, *x*_*n*_ represents feature of facial image (I) and *y*_*i*_=1,0 of class labels of male and female, respectively; the separation hyperplane using a linearly separable binary classification problem is given by 15. For nonlinearly separable data, input space can be mapped to high dimension feature space using other kernels as RBF. Here, a soft margin SVM is used, and hyperplane can be found by inducing a slack variable, allowing some errors at the training time. The problem of finding optimal separation hyperplane by reducing the training error is denoted as

(15) $$\begin{array}{c} \begin{array}{c} \text{minimize}=\frac{1}{2}w^{T}w+C\overset{N}{\underset{i=1}{\sum }}\xi_{i},\\ \text{subject to}:Y_{i}\left(w.\phi \left(x_{i}\right)+b\right)\geq 1-\xi_{i},i=1,2,\ldots ,N,\\ \xi_{i}\geq 0,\;i=1,2,\ldots ,N. \end{array} \end{array}$$

Here, *w* is the weight as usual to the maximum separating hyperplane, and *b* is the bias. Cost parameter C determines the trade-off between the distance of hyperplane and training error. For different nonlinear problems, different types of kernels can be utilized [46]. In our experiments, two kernel functions, linear and radial basis function (RBF), are used.

### 3.6. Naive Bayes Classifier

For a given image set *I*_*d*_={*i*_1_, *i*_2_,…, *i*_*n*_} and feature set *X*_*i*_ for *i*^*th*^ image, we have to decide the probability for each class *y*_*i*_=*c* in *Y*={*y*_*i*_,…, *y*_*n*_} (male and female). Equation (16) shows the class marginal probability [47]:

(16) $$\begin{array}{c} \begin{array}{c} p\left(y_{i}=c|I_{i}\right),\theta \propto \theta_{c}\Pi_{n}^{D}p\left(i_{mn}|y_{m}=c\right)\\ \propto \theta_{c}\frac{\sum_{n}i_{mn}!}{\left\{i_{m1}!,i_{m2}!,\ldots ,i_{mD}!\right\}}=\Pi_{D}^{n}\theta_{nc}^{mn} \end{array}. \end{array}$$

Data likelihood is represented in the following equation:

(17) $$\begin{array}{c} \begin{array}{c} p\left(I,Y\right)=\Pi_{m}^{N}p\left(y_{i}=c|i_{i},\theta \right)=\Pi_{m}^{N}\\ \Pi_{c}^{C}\theta_{c}^{1\left(y_{m}=c\right)}\Pi_{n}^{D}p{\left(i_{mn}|y_{m}=c\right)}^{1\left(y_{m}=c\right)} \end{array}. \end{array}$$

### 3.7. Logistic Regression

In this paper, a Logistic regression classifier is used, forming a nonlinear relationship between training instances and their known labels and has a distinct cost function. If gender class male is represented by (*c*=1) and female by (*c*=0), probability (P) for male on the basis of observed feature vector *X*=[*x*_1_,…, *x*_*n*_] is given by the equation as follows [48]:

(18) $$\begin{array}{c} P_{i}\left(C=1|X_{i}\right)=\hat{y_{i}}=\frac{1}{\exp^{-\left(w_{1}x_{1}+\cdots +w_{n}x_{n}+b\right)}}. \end{array}$$

Here, *b* is the intercept or bias, and [*w*_1_,…, *w*_*n*_] are the weights or coefficients associated with features [*x*_1_,…, *x*_*n*_] ∈ *X* (features vector), respectively, for the input image *I*.

## 4. Experiments and Result Analysis

The experiments of the proposed architecture for facial gender identifications are carried out on the adult face and juvenile faces (immature minor age) separately. We have used 14,051 images of the FERET dataset (adult age from 0 to 70 years), and 20,000 images from the UTK Face dataset. Each dataset is divided into two disjoint training and testing sets with the ratio of 80/20 for five-fold cross validation (K5). We have performed multiple experiments to find Gabor's optimization and verify that the proposed feature engineering is the best among Gabor, Gabor-DWT, Gabor-Mean, and proposed Gabor-meanDWT. For this, the mean accuracy (*A*) (as shown in (1)) is evaluated with different feature engineering techniques such as Gabor, Gabor-DWT, Gabor-Mean, and proposed Gabor meanDWT, which is shown in Table 3. It is evaluated with a classifier support vector machine (SVM) for FERET and UTK Face datasets. The detailed analysis is discussed as follows:

### 4.1. Feature Size

The original input face of any dataset is passed from the PCN face detector, which provides facial points on the input image, and the respective face is cropped after detection using the PCN face detector. These cropped faces are subsequently rescaled in a uniform resolution of size 128*∗*128*∗*3 and converted in greyscale (prepossessing). The feature engineering is applied to it, which generates a feature vector. Gabor generates 368640=(128*∗*128)*∗*5*∗*(0.25*∗*0.25+0.5*∗*0.5+1*∗*1+1.25*∗*1.25+1.5*∗*1.5) for 128*∗*128*∗*3 with 5 orientations (*θ*) and 5 scales *s*=0.25, 0.5, 1,1.2581.25. Gabor-DWT with three levels and Gabor-mean generate the 6560 and 83968 features, respectively, while the proposed Gabor-meanDWT generates least/compact feature vector size of 1,312 which is presented in Table 3. This step is repeated for all the images (training and testing) of both datasets. The compact size of the feature vector reduces the timing complexity of the prediction of gender significantly. Thus, the proposed technique takes less time in convergence and inference from the trained model results. The small feature size makes the proposed approach an appropriate choice with edge device (Acorn RISC Machine (ARM)-RISC architecture) where memory constraint is a significant issue. Thus, the proposed architecture of gender recognition can be used in ARM-based real-time devices such as CCTV cameras, mobiles devices, to make them smart with key decisions using the face.

### 4.2. Accuracy

From the analysis of results presented in Table 3, it is concluded that the proposed architecture with Gabor-SVM, Gabor-DWT, Gabor-mean, and Gabor-meanDWT with SVM-Linear kernel achieved an accuracy of 90.2%, 91.5%, 94.55%, and 99.9% on the FERET dataset (adult faces). The proposed Gabor-meanDWT feature extraction technique achieves the best accuracy on adult faces (FERET data) and juvenile faces (UTK Face) because the proposed steps of the mean feature extraction technique remove the nondiscriminatory redundant features effectively from Gabor matrices, as shown in Figure 5. The accuracy of the proposed Gabor-meanDWT is far better than with the existing state-of-the-art techniques which are shown in Table 2 as Gabor-fuzzy [16], LBP [27], and CNN based technique [38] for adult faces and juvenile faces [32]. The Gabor-fuzzy got 98% accuracy on adult faces, while the proposed approach achieved 99.9% accuracy. The proposed architecture detects the oriented faces correctly, which provides a significant contribution to accuracy also. Thus, high accuracy with low memory consumption makes the proposed approach for various use cases of real-time application on edge devices.

The proposed technique archived better accuracy compared to most of CNN-based techniques as 98.9% of VGG-16 [31] on adult faces and [34–36] on juvenile faces, as shown in Table 2.

The limitation of the proposed work is that its accuracy is lesser than the accuracy of the ensemble approach of ResNet [38] on juvenile faces, but the ensemble approach calls multiple layers of the classification model, which takes a vast amount of computation resources and data during training and regularization. The proposed approach is based on conventional learning, which takes limited resources of computations and is regularized on limited data.

### 4.3. Juvenile v/s Adult Faces

For adult faces, the proposed model Gabor-meanDWT achieved 99.9% five cross-validation accuracy on real-life adult faces of the FERET dataset using SVM-linear kernel classification. In contrast, the accuracy on the juvenile faces of the UTK Face dataset is 95.5%. The other methods such as Gabor, Gabor-Dwt, and Gabor-mean show the same trends of pattern for the FERET and UTK datasets. The decrease in accuracy for the UTK dataset is due to immature and nondistinguishable features on juvenile faces of UTK compared to FERET. Moreover, the UTK dataset has more complex real-time challenges such as background change, illumination, POSE (position, orientation, scale, and expression variations), and head orientations than the FERET dataset. The proposed solution provides robust results over the varying age even with unconstrained environments (face rotation, illumination, etc.), making it a better choice for various industrial real-time applications.

The juvenile face has lower accuracy than adult faces because the stress on soft tissue shows as an initial sign of aging in teenagers. Adult aging is affected by morphological changes in wrinkles, skin textures, and facial lines on the forehead with different horizontal and vertical shapes. The size of the face grows with age. It is shown that the performance of facial gender recognition degrades on child's faces compared to adult faces [49, 50]. So, the proposed technique on juvenile faces has lower 95.5% than 99.9% accuracy on adult faces.

### 4.4. Comparison for Different Classifiers

**Figure 7:**
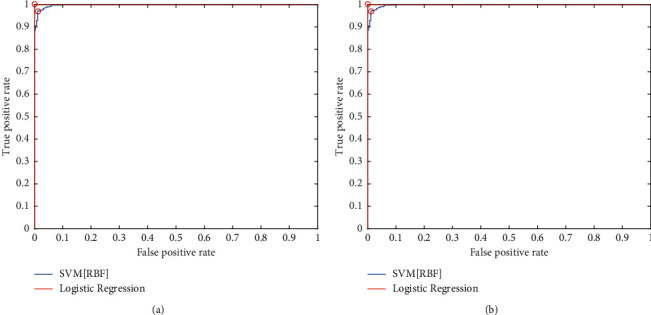
ROC Curve of proposed gender recognition model (Gabor-MeanDWT). (a) Classifier: SVM (RBF), Logistic Regression and (b) classifier: SVM- Linear and Naive Bayes; Protocol: five cross validations (K5); Dataset: FERET.

The proposed Gabor-meanDWT feature engineering architecture of gender recognition is converged and experimented with different classification techniques, including SVM-linear, SVM-RBF, Naive Bayes, and linear regression. The receiver operating characteristic curve (ROC) is evaluated for the proposed architecture with four classifiers.  Figure 7 shows the receiver operating characteristic (ROC) curve for the proposed feature engineering model with SVM (RBF), logistic regression and SVM (linear), and Naive Bayes classifier, respectively, on the FERET dataset.  Figure 7 represents that area under the curve (AUC) for SVM-linear is more than the AUC for SVM with RBF kernel because features are linearly separable. The area under the curve (AUC) and optimum points (OP) of the ROC curve for the proposed model with different classification techniques are evaluated, which is shown in Table 4. The best optimum point is achieved with SVM-linear and Logistic regression, while the SVM (RBF) kernel achieved 97.07 true positive rate (TPR) on 0.012 false positive rate (FPR) with AUC of 0.998. The confusion matrix of proposed Gabor-meanDWT feature extraction is also evaluated for UTK Face data with linear SVM and RBF SVM, which is shown in Table 5. The results show that male data is less confused than female because male faces have unique or discriminating features of the face compared to female faces. Thus, face discrimination also affects the accuracy of a particular gender recognition system.

## 5. Conclusions

The proposed architecture initially detects the invariant orientation faces using the PCN network and then proposes the Gabor-meanDWT technique to extract the illumination of invariant features with the reduction in redundancy and dimensions to converge the learning of the classifier network effectively. Edge and texture features are computed by applying a scaled Gabor filter using five orientations and five scales. It produces a 25 Gabor bank matrix with some common redundant features reduced by evaluating the average value of the respective Gabor coefficient and orientation. The average features smoothen the edge-texture features and reduce the dimensions by a factor of orientation (here, it is reduced by 1/5). Subsequently, the discrete wavelet transform (DWT) feature extraction technique with derived Gabor-mean features converts it into four frequency domains, including low frequency to high frequency. The smooth part of extracted features is represented by the LL sub-bands of DWT, which have the size of 1/4 of its source input. This process generates a compact, concise, and unique pattern representing the gender class ‘boy' or ‘girl'. The optimized features are trained with one of the classifiers (a) SVM-linear, (b) SVM-RBF, (c) Naive Bayes, and (d) logistic regression. For regularization and unbiased results, mean accuracy is evaluated with five-fold cross validation. The training testing ratio is 80 : 20 on the FERET and UTK datasets, respectively. The results of the proposed model are shown in Table 3. Table 3 shows that the proposed model achieves the highest accuracy of 99.9% with SVM (Linear kernel) on adult faces (FERET dataset) and 99.5% on child's faces (UTK dataset). The UTK dataset shows lower accuracy due to immature features on child's faces and its challenging uncontrolled environment compared to the FERET dataset. SVM with RBF achieved AUC=0.998 (Area under the curve), as shown in Table 4, while SVM-linear kernel shows highest AUC=1 which outperforms the others.

The proposed approach of facial gender recognition has consistent accuracy for real-time use cases, including the head orientation, illumination variations, and over the age variation persons (age invariant). However, it is a limitation to test with a small image (far distance objects from camera sensors), dark image (image generated in low light visibility), different continental/race persons. These factors can be tested and improved by researchers in the future.

## Figures and Tables

**Table 1:** Publicly available dataset for gender recognition.

Dataset	Images,	Age range	Environment
	Subjects	(Years)	Challenges
UTK Face [8]	20 000	0 to 116	C, I, E, L, O, F, s

MORPH-II [9]	55 134	16 to 77	C, L, R, H
	13 618		

LFW [10]	13 233	—	U
	5749		

FG-NET [11]	1002,	0 to 69	R, I, E, O, S, s
	82		

FERET [12]	14 051	10 to 70	C, P
	1199		

IMDB-WIKI [13]	523,051	0 to 100	U
	20 284		

Adience [14]	26 580	0 to 100	U
	2284		

CelebA [15]	202 599	2 to 18	C, I, E, L, O, F, s
	10,177		

The above table describes different datasets with a size of dataset including the number of faces, subjects, age range (in years), and controlled variations such as Resolution(R), Illumination/Lighting (I) Sharpness (S), Expression (E), Race (R), Occlusion (O), Hair (H), Frontal View (F), Scale (s), Profile (P), Longitudinal (L), Controlled environment (C), and Uncontrolled environment (U).

**Table 2:** Different state-of-the-art techniques including featuring engineering and classification methods for facial gender recognition for FERET (adult faces) and UTK face dataset (juvenile faces).

Dataset	Authors	Feature extraction	Accuracy
		Classification	(%)
FERET	Leng and Wang [16]	Gabor-SVM, Fuzzy	98.0
	Rai and Khanna [17]	Gabor-2DPCA-SVM	98.2
	Aroussi et al. [18]	DWT-SVM	92
	Mohamed et al. [19]	DCT, DWT-SVM	95
	Wang et al. [20]	Gabor, SIFT -AdaBoost	97.0
	Ozbudak et al. [21]	DWT-PCA-FDA	9
	Lu and shi [22]	2D PCA-SVM (RBF)	94.8
	Bissoon and Viriri [23]	PCA-LDA	85
	Jain and Huang [24]	ICA-LDA	99.3
	Tapia and Perez [25]	LBP + Intensity+	99.1
		Shape-SVM	
	Makinen and raisamo [26]	LBP, Haar-ANN, SVM	92.9
	Alamri et al. [27]	LBP, WLD-N.Neighbor	98.8
	Moeini et al. [28]	LGBP-SVM	98.5
	Patel et al. [29]	CoLBP-SVM	93.9
	Annalakshmi et al. [30]	SLBP + HOG-SVM	97.6
	Aslam et al. [31]	CNN (VGG-16)	98.9
	Afifi and Abdelhamed [32]	Foggy face-Deep CNN	99.3

UTK	Swaminathan et al. [33]	Face Embed (FE)-SVM	88.4
		FE-logistic regression	92.4
		FE-naive-bayes	89.4
		Fe-KNN	97.0
		FE-decision trees	93.2
	Teru and Chakraborty [1]	CNN	89.5
		CNN-WL (weight loss)	88.8
		Fader CNN	84.8
	Song and Shmatikov [34]	CNN	90.4
	Bragman et al. [35]	CNN	92.5
	Nagpal et al. [36]	CNN	94.6
	Das et al. [37]	MTCNN	98.2
	Abdolrashidi [38]	Ensemble of ResNet	96.5

AD	Hassner et al. [39]	LBP + FPLBP-SVM	79.3
	Khan et al. [40]	PCS-RDF	91.4

FG-NET	Nayak and Indiramma [41]	PCA	61.13

**Table 3:** Comparative results of the proposed model for (a) Gabor, (b) Gabor-DWT, and (c) Gabor MeanDWT.

Dataset	Feature Selection	Number Features	Classifier	Accuracy
FERET	Gabor	419 840	SVM (L)	90.2%
	Gabor-DWT	6560	SVM (L)	91.5%
	Gabor-mean	83 968	SVM (L)	94.55%
	Gabor-meanDWT	1312	SVM (L)	99.9%

UTK	Gabor	419 840	SVM (L)	83.45%
	Gabor-DWT	6560	SVM (L)	89.45%
	Gabor-mean	83 968	SVN (L)	90.2%
	Gabor-meanDWT	1312	SVM (L)	95.5%

The Gabor MeanDWT method outperforms both Gabor and Gabor-DWT and other state-of-the-art methods, as shown in Table 2.

**Table 4:** The optimum point (OP) of receiver operating characteristic curve (ROC) and area under the curve (AUC) for the proposed feature engineering model (Gabor-meanDWT); Protocol: five cross validation (K5); Dataset: FERET; Classifier: SVM-RBF, SVM-Linear, Naive Bayes, and logistic regression(LR), respectively.

Classifier	Gabor-meanDWT	
	AUC	Optimum Point (FPR, TPR)
SVM (RBF)	0.998	0.012, 0.970 7
SVM (Linear)	1	0,1
LR	1	0,1
Naive Bayes	0.767 0	0.588, 0.946

**Table 5:** Confusion matrix of proposed Gabor-meanDWT feature extraction using UTK Face data.

A.SVM (linear)			B.SVM (RBF)		
	Female	Male		Female	Male
Female	89%	11%	Female	98%	2%
Male	5%	95%	Male	1%	99%

The results show that male data are less confused than female.

## Data Availability

The dataset used for experiments is publicly available on the given citation. The proposed design, results, and investigations used to support the findings of this study are included within the article and cited appropriately. The software code written for the experimental used to support the results of this study is copyright of the authors and so cannot be made freely available. Requests for access to code-related query should be made to Sandeep K. Gupta (email id: sandeepmbm@gmail.com).
